# The Nursing Supplement

**Published:** 1888-06-23

**Authors:** 


### The Nursing Mirror.

All Contributions for this Supplement should be addressed "The Nursing Mirror," care of The Editor, 38, Old Jewry, E.C.

### En Passant.

LADY GUARDIANS.—There were two meetings held last week to promote the return of women as Poor Law Guardians, a fact which will interest our infirmary nurses. But even a lady guardian cannot understand the necessities of nursing without ever having had experience as a nurse, so that another proposal has been made by Miss Twining before the House of Lords Committee. This is that a trained nurse should be appointed inspector of nursing in workhouse infirmaries, and should visit all State hospitals and see thoroughly how the nursing is being done, and point out all faults and declare the proper remedies. The proposal is simply admirable, and what a pleasant post would that be of "Inspector of Nursing."

PROGRESS IN PARIS.—The women are coming to the front more than ever in France. There are lady doctors, lady stockbrokers, lady reporters, and, recently, a lady defended her father in a law-court with acumen worthy of an ancient forensic hand. Now the Sorbonne has awarded for the first time to a woman a diploma of *Docteur-ès-Sciences*. The recipient of this honour is a modest young lady, named Mdlle. Leblois, daughter of a Strasbourg pastor, and she is described as a veritable prodigy of learning, being as clever at the Differential-Calculus as some of her sisters are at the lighter and gayer science of coquetry. Mdlle. Leblois was loudly applauded when her name was read out for the exceptional prize which she has gained for her remarkable erudition.

SECTARIAN SYSTEMS OF NURSING.—On Hospital Sunday there was an attempt made to revive the old discussion about the employment of nursing sisterhoods in the metropolitan hospitals. One minister refused to hold the special collection on Hospital Sunday, preferring to have it another day, when the fund could not claim the money; and it could all be bestowed on some hospital with an unsectarian system of nursing. The minister in question labours under a misapprehension, as no London hospital has a sectarian system of nursing, and so he protested over much. Nothing could be fairer or better than the plan upon which nurses are engaged at the overwhelming majority of the voluntary hospitals throughout this country. None can deny that sisterhoods do their nursing work thoroughly and well, and so long as this is so, none can expect the hospitals to dispense with their services.

PREACHERS ON NURSES.—In the special sermons preached on Hospital Sunday there were several references to the good work done by nurses. The Rev. C. F. Dermer, at the morning service at Westminster Abbey, remarked "that against the tales of common cruelty, of widespread class selfishness to be seen week by week, were these truly wonderful acts of noble philanthropic lives to be found on all hands among Christian men and women working in the name and for the love of Christ." The Rev. Dr. Wace practically observed: "The funds received are administered with due economy, and there is no class of workers who labour for less pecuniary reward than the sisters and nurses who have the more immediate care of the sick in the hospitals." Dr. Vaughan, preaching to a crowded congregation, said: "Refined and delicate women are consecrating themselves into ministering angels beside the beds of harrowing and repulsive disease."

CROYDON PALACE.—The beautiful old palace of the Archbishops of Croydon has been bought and presented to the Kilburn Sisters. This ancient building has always been a religious house, but lately has been crumbling into ruins, and it seemed possible that one more landmark monkish days was to vanish. Now, thanks to the generosity of a noble donor, the palace is to become a country retreat and home of rest for the Kilburn Sisters, where they can recuperate their strength, and enjoy repose when worn out with their labours amongst the London poor and sick. On July 5th a bazaar is to be held in the Palace grounds, to gather funds towards the much needed work of restoration.

HOSPITAL OUTLINES.—Mr. W. C. Henley, whose sketches and portraits of hospital life appeared in the "Cornhill" in 1875, has published a book of verses which contains many of his early efforts, improved and completed. We quote some of his lines on a staff nurse, and on a lady probationer:

"The supreme poets of the commonplace,
George Eliot and Rembrandt—only these
Could paint her all to you: experienced ease,
And antique liveliness and ponderous grace;
The sweet old roses of her sunken face,
The depth and motive of her shy grey eyes.

"This is her picture—seven and thirty years;
A Roman nose, a dimpling double chin,
And dark shy eyes, if ignorant of sin,
Not unacquainted, it would seem, with tears;
A comely shape; a slim, high-coloured hand,
Graced, rather oddly, with a signet ring;
A bashful air, becoming everything;
A well-bred silence always at command."

PATIENTS AND NURSES.—Under the above heading the *Daily News* had an article on the conversational powers of nurses. It maintained that nurses are never weary of relating what doctors call "cases," and love to dwell minutely on the particulars of accidents and operations. The more horrible their tales and the greater their effect on the patient, the more pleased are the nurses with their powers of narration. Of course this accusation can only be brought against a small portion of a noble profession, for certainly the many highly-educated women who have lately taken to nursing, have other topics of conversation besides themselves and their work. There is probably a grain of truth at the bottom of the statement, for nursing is a most absorbing and interesting profession, and a nurse in a hospital lives in a little world of her own. Few outside friends penetrate into hospital wards, and common interests cause warm friendships to be struck up between nurses. They naturally discuss their duties and their patients to a large extent; there is so much to learn and such interest to them in the smallest details, that they are indeed apt to get into a narrow groove of thought, and let empires rise and fall without heed. Often a matron has to complain of the difficulty of persuading her nurses to seek proper recreation; and then, when these women emerge from their comparative seclusion, and go into private families, they find they have lost touch with the passing topics of the day, which form the lightest and least exciting conversation for their patients. Truly the demands upon a nurse seem endless, and yet it is evident that an attendant with a cultivated mind can best brighten and amuse the weary hours of sickness. Amongst all your other duties, oh ministering women, you must yet find time to keep yourselves acquainted with passing events and the waves of contemporary thought.

### Original Articles.

### A NURSES' PROVIDENT FUND.

There is an institution for nurses at Lincoln, in what is known as the "White House," where they have an excellent Provident Fund, in which all can invest their savings. There are nearly 50 nurses belonging to the institution, and they receive from £14 up to £30 a-year, according to the length of time they remain at the White House. Everything is provided for them, including uniform and medical attendance; so that all ought to be able to invest part of their salary yearly, and those who do put their savings into the fund receive the following privileges :—

For the first term of three years of her service a nurse may invest any sum not exceeding £2 in each year.

For the second term of three years, any sum not exceeding £4 in each year. If she leaves the institution at the end of these six years, she will be entitled to receive back the sum she has invested, with an addition of a bonus of 50 per cent.

| Example— | £ | s. | d. |
|---|---|---|---|
| First 3 years at £2 each year | 6 | 0 | 0 |
| Second 3 years at £4 each year | 12 | 0 | 0 |
| | 18 | 0 | 0 |
| 50 per cent. bonus | 9 | 0 | 0 |
| | £27 | 0 | 0 |

If she remains at the institution she may continue investing any sum not exceeding £4 in each year, until the completion of her ninth year of service, and if she leaves after these nine years, she will be entitled to a bonus of 75 per cent. on the sum invested.

| Example— | £ | s. | d. |
|---|---|---|---|
| Three years at £2 | 6 | 0 | 0 |
| Six years at £4 | 24 | 0 | 0 |
| | 30 | 0 | 0 |
| 75 per cent. bonus | 22 | 10 | 0 |
| | £52 | 10 | 0 |

If she still remains at the Institution, she will be allowed to invest any sum not exceeding £6 in each year, until the completion of her twelfth year of service, and on leaving, at the expiration of these twelve years, she will be entitled to a bonus of 100 per cent. on the sum she has invested.

| Example— | £ | s. | d. |
|---|---|---|---|
| Three years at £2 | 6 | 0 | 0 |
| Six years at £4 | 24 | 0 | 0 |
| Three years at £6 | 18 | 0 | 0 |
| | 48 | 0 | 0 |
| 100 per cent. bonus | 48 | 0 | 0 |
| | £96 | 0 | 0 |

The bonus is not given to any nurse who leaves the institution under six years, and it will be seen that the whole scheme is carefully arranged, so that those who remain most steadily at their posts receive the greatest benefit. If a nurse is dismissed for misconduct, she also loses all claim to any bonus. The nurse who remains faithful to her duty at the White House for the twelve years, and invests one-sixth of her earnings during that time, receives on departure exactly double the sum she has saved, and walks away with nearly £100 in her pocket. Of course, there are some people who would call this degrading charity, and others who would term it bribery, but the chief persons concerned are the nurses; and if they pay into the fund, and the institution thinks well to provide the bonus, the two contracting parties are satisfied, and the public can say what it will. It is sad that women need to be tempted to save by the gift of bonuses, and that ordinary rates of interest will not satisfy their unbusinesslike minds; but the habits of generations are binding and hard to break free from, and so long as women's wages are abnormally small, they will expect the interest on their savings to be abnormally large. It is not only the country institutions which find it necessary to aid their nurses in providing for the future—many of the best known hospitals do the same. At Guy's there is a provident fund, which gives what might be termed a charitable rate of interest, which practically amounts to pensioning off those who grow gray in their service. In every department of the nursing world we see the necessity for helping nurses to lay by for a rainy day, and much though we may regret that they cannot provide for their future without extraneous aid, we are obliged to acknowledge that the want is there, and must be met.

### Notes from America.

"Life's Work Well Done."—We have to record with great regret the death of Miss Alice Fisher, lately matron of the Blockley Hospital, Philadelphia. Miss Fisher was the daughter of the Rev. George Fisher, and was trained at the Nightingale School, St. Thomas's. While there she had an attack of acute rheumatism, which laid the foundation of the heart trouble that finally ended in her death on Sunday, June 3rd. After acting some time as assistant superintendent at the Edinburgh Infirmary, Miss Fisher was appointed matron of the Newcastle Hospital, and afterwards superintendent of "Addenbrooke's," Cambridge, where she remained five years. From there she went to Radcliffe Infirmary, Oxford, and later on to the Birmingham Hospital, and lastly to Philadelphia, to become matron of the "Blockley." Under her management the Philadelphia Hospital has been completely reformed, and is now considered to be one of the finest in the world. Her services have not been entirely confined to Philadelphia. After her arrival in America there was an outbreak of typhoid fever at Plymouth in Pennsylvania, in 1885. She applied for, and obtained leave of absence "for a holiday," the whole of which time she spent in organising the hospital service there. This self-imposed duty occupied two months. Her last illness extended over a year. Her desire was "to live that she might continue her usefulness." During the last week of her life her late assistant, the wife of Senator Hawley, had the sad satisfaction of ministering to her necessities and hearing her last wishes, one of which was that her remains be laid in a spot, chosen by herself, in the Woodlands Cemetery, because it had a commanding view of "Blockley." The following is from the *Philadelphia Press*: "Dr. J. William White, who had seen Miss Fisher daily for many months, who was her devoted friend and adviser, and who was interested in all her plans for the improvement of nursing in Philadelphia and at Blockley, when seen in reference to her death, said that the city had suffered a loss which was irreparable. Others, he said, there might be who would carry on in the directions indicated by her the beneficial work she had begun, but there could never be another who combined her energy and unswerving strength of purpose with the personal magnetism and sweetness and gentleness of character which made her loved as well as respected. Her work was extraordinary, both in its quantity, which would have worn out many strong men, and in its quality, showing a masculine force and breadth of understanding with a feminine tact and insight into character which made her one of the moving forces of any community in which she lived, and justifies, in his opinion, the statement that she was one of the remarkable women of her time. As to the loss felt by her personal friends, that could not be expressed in words. Her place in their hearts can never be filled."

### The Nurses' Bookshelf.

### Hospital Sisters.*

The second edition of this useful work on "Hospital Sisters and their Duties," by the matron of the London Hospital, merits a most cordial welcome. Miss Eva Lükes is universally recognised as one of the ablest gentlewomen who have devoted themselves to nursing the sick. Her position as the head of the nursing staff of the largest hospital in this country is unique, whilst her sober judgment, her exceptional capacity, and her grace of manner, have made her all-powerful in the nursing world. Small wonder then that her book possesses great practical value for all connected with nursing; or that it includes much that probationers, nurses, and matrons can learn, besides the special directions to sisters.

The introductory chapter takes a very high view of the standard at which a sister ought to aim, and paints an ideal which is almost depressing in its beauty. But, as the author herself says, it is better to work towards an ideal that may be impossible of actual attainment, than to contentedly accept our duties as mechanical, and not see the wide possibilities which lie before us. While recognising the strain on a hospital sister who truly fulfils every duty, Miss Lückes is anxious to impress on the newly-made sister that worry will not help her through her work, and that if she will patiently accept things as they are for a short time, her after alterations in the plan of the day's work, etc., will probably be more enduring. To stand in a position of authority for the first time is always trying, but gives a wider influence into a woman's hands. A sister should be capable of regarding her post from the standpoint of an outsider; and, looking beyond her own institution, should betray a universal interest in all subjects connected with hospital life.

The second chapter deals with the domestic management of the ward, and the supervision of the ward-maid. To the public it may seem strange that a sister should be urged to daily note how the floor is scrubbed, and by continuous supervision ensure the cleanliness of refrigerator, scullery, kettles, etc. It rests with the sister also to allot the ward linen, to see that it is aired and repaired, and that all which is beyond mending is used as floor-cloths and dusters. All the routine work which the outsider might imagine did itself is kept going by the sister, who, if she is a capable manager, will yet keep all in order without expending much thought on small things. The third chapter defines the relationship of sisters to their staff nurses; a most difficult subject, so nearly does the work of both run in the same line. A staff nurse often has had great experience, and is well up in all nursing particulars; it then becomes a temptation to a busy sister to leave details of management to her capable subordinate, which really belong to her department. The author is very anxious that sisters should not fall into this error, and, by shirking their responsibilities, lower their power of usefulness. The small hints as to where and how a sister should assert herself must be useful to many, clearing up small points which are apt to remain doubtful, and be the beginning of greater evils. The sister is to be very strict in procuring the staff nurses' recreation, and also in protecting the probationers from the bullying which they often have to endure at the hands of the experienced nurse.

The next chapter deals entirely with the training of probationers, and the wonderful sympathy which Miss Lückes shows with all the small difficulties which beset the new candidate in the nursing world, betrays a mind of vast power for feeling for others. Few who have become matrons of large establishments appear to remember the trials of their probationer days, and those whose memories are good too often think that because they endured hardships others should do the same. A constant succession of ignorant probationers must be enough to try any woman's temper; but Miss Lückes reminds her readers that those who cannot impart their knowledge may be good nurses, but are utterly unsuitable to become sisters. Only by thoroughly teaching all details to new probationers can an efficient staff of nurses be maintained in a hospital; and it is on a sister's careful observation of the capabilities of her pupils that they are either advanced or otherwise. Considering that some of the best physicians and surgeons of the day lecture year after year to the new probationers, it will be seen that this turning of raw material into skilled nurses, though trying work, is of great consequence, and one which no sister can dare to shirk. The relationship of sisters to their patients, which is explained in the fifth chapter, is a subject of such extreme importance there is no fear of its ever being overlooked. Yet there are many small courtesies pointed out, such as welcoming new patients, and listening patiently to their friends, which are often forgotten. The hospital provides all comforts for the patients, and it is for the sister to dispense these hospitalities in a pleasant manner. The ideal sister will see that dominoes or draughts are provided for the cheerful, and books for the studious; that every facility is given to the entrance of friends of those who are very ill; she will even give every patient an opportunity of unburdening his mind to her, and her Protean nature will sympathise with all the remarkable and varied temperaments of those she attends.

Alas! surely it is all an ideal? The public who have so often been made to feel intruders on entering a hospital to visit a friend, will wonder where the author ever saw the type she holds up as a pattern. The sister, as painted by Miss Lückes, is too like a heavenly vision, and far removed from the earthly reality. And yet if anywhere we are to find women of nobility above the average, it is certainly amongst our hospital sisters. They have remained true to a trying profession throughout the wearisome period of probation, and have deliberately taken a responsible post amongst the most heartrending scenes. With open eyes they have accepted a life of labour, they have proclaimed to all the sick and suffering, "Lo, I am your sister; my lot is cast in your ways." And to these women to have such practical yet high-minded counsels given them as are contained in this book, must indeed be a help and a comfort. The small details, which soon grow dreary, are here shown in a new light; the little difficulties before which many give way because they are not worth fighting against, are here clearly pointed out and proclaimed worth overcoming. Every hospital sister should possess this book, and every nurse and probationer can here learn much never taught elsewhere.

* By Miss Luckes, Matron to the London Hospital; Author of "Lectures on General Nursing." London: J. and A. Churchill. Price Halt-a-Crown.

### Vacancies.

The following vacancies are announced:—

**Lady Superintendents.**

The Sanatorium, Hull; £70. Evesham Sanatorium; £30.

**Sister.**

Royal Hospital for Children and Women; £30.

**Matron.**

Swindon Victoria Hospital.

**Nurses.**

Miller Hospital, Greenwich; £22.

Northern Convalescent Fever Hospital; £30.

Glasgow Sick Poor Nursing Association.

Southport Trained Nurses' Home. Several.

Hoxton House Asylum.

Chelsea Hospital for Women; £20

Workhouse Infirmary Nursing Association.

Poorhouse Hospital, Irvine; £25.

District Nurses' Home, Lovell Street, Leeds.

### Everybody's Opinion.

[*Correspondence on all subjects is welcome, but correspondents would oblige by writing on one side of the paper only.*]

### THE PAY OF PRIVATE NURSES.

Having read "H. J.," matron's question in The Hospital of the 12th of May in reference to the percentage of private nurses, I think it only fair that nurses should receive a little extra in cases of heavy nursing. For instance, in mental, infectious, and other cases, where the fees are double, nurses work harder and run greater risk, while the institutions are the gainers. Every nurse in private nursing knows how hard they sometimes have to work. For instance, a nurse goes to two cases in one week sometimes, after perhaps losing several nights' rest; she cannot always adhere to the rules of the institution to which she belongs as regards sleep, etc.; she is often thrown among ignorant people who are not competent to look after her charge while she is asleep, or perhaps the family are already worn out and cannot help. I have myself left a mental case and immediately gone to an infectious case (which died on my arrival), come home, and gone to another, so commenced my third two guineas between Saturday and Monday. I may as well here mention that I was at the mental case twelve weeks, bringing in twenty-four guineas. Another time I was nursing a farm-servant with one of the worst cases of erysipelas I ever saw (and I have seen many); there were two rooms in the house, one up and one down, and as there were three in family, there was no room for me; both parents were very old, and although good old people, were not very intelligent; lodgings were got for me at the inn about half-a-mile from the hut where they lived. Suppuration was rapidly taking place, and poultices were changed every hour or so. I could only take a few hours' rest every second night, going to the inn before closing time, and then I have been fetched because bleeding has commenced and they have been frightened, and so I worked for three weeks, earning two guineas, while my salary was £20 a-year. I know all cases are not so hard, but we earn money just the same, whether the work be light or heavy. When one is young one does not mind it so much, but after a few years it tells on one's constitution and makes one think of the future; besides, nurses have other calls sometimes on their incomes. My readers must not judge me unkindly or think me mercenary, but in every other vocation one looks for a rise, and why should nurses not? If my readers think me un-nurselike or wanting in dignity, let them think that institutions ask for money—why not we? Islands.

### A BRAVE HOSPITAL NURSE.

Having seen the paragraph in last week's Hospital under the above title, we feel that to young nurses especially, a word of caution is due. Such an act as is there spoken of is looked upon by most of the medical and nursing world as an unjustifiable risk to life, and has, on more than one occasion, met deservedly, not with praise, but with grave censure. It is not the record of such deeds as this which "keeps bright our enthusiasm," but the knowledge that there are hundreds of nurses performing amid their daily duties many unrecorded acts of real heroism, and "absolute unselfishness."

Two Sisters.

### A PRACTICAL EXPERIENCE.

It may be useful to younger nurses to know that glycerine will not always act when given as an injection. I was sent for at ten o'clock at night to give a soap enema, which had been ordered by the doctor, and had been twice given by the friends, without success. I took with me some glycerine and a small glass syringe, and gave a teaspoonful immediately, waited half-an-hour, I then sent for fomentation flannels, when a message came from the doctor: "Give glycerine and foment." I then gave twice the quantity of glycerine, fomented, and waited again half an hour; not succeeding, I gave a soap injection, and lastly, three-quarters of a pint of linseed oil. Fomentations continued, and at three o'clock the patient had relief. District Nurse.

### SIR FREDERICK LEIGHTON'S COUNSEL.

The following lines by Sir Frederick Leighton are so encouraging to myself and many of my fellow workers, that I am sending them to you for the N. M., trusting other nurses may read them: "Whatever of dignity, whatever of strength we have within us, will dignify and make strong the labours of our hands. Whatever noble fire is in our hearts will burn also in our work. Whatever purity is ours will chasten and exalt it, for as we are, so our work is, and what we sow, that, beyond a doubt, we shall reap, for good or for ill, in the strengthening or defacing of whatever gifts have fallen to our lot." N. E. M.

### Appointments.

[It is requested that successful candidates will send a copy of their applications and testimonials, with date of election, to The Editor, The Lodge, Porchester Square, W.]

Miss A. Mawer has been appointed matron of the Hangleton Hospital for Infectious Diseases, Hove. Miss Mawer was trained at the Seamen's Hospital, Greenwich, and for the last year has acted as Resident Superintendent of the Evesham Sanatorium. Miss Mawer's kindness and attention to the patients at the Sanatorium, and her decided aptitude for the responsibility of administration, gained high commendation from the Medical Officer of Health, and her departure from Evesham will be regretted.

Miss Alice G. Howard Jones has been appointed matron of the Central London Ophthalmic Hospital. Miss Howard Jones was trained at St. Bartholomew's, where, both as probationer and as staff nurse, her skill, tact, and sympathy attracted the attention and won the approval of the physicians there. Since completing her training, Miss Howard Jones has undertaken various holiday duties; and among others, acted as matron of the Grosvenor Hospital for Women and Children for a period of nine months, where she proved herself fit for a responsible position, and competent to instruct and guide others in the task of nursing the sick. We congratulate the Central London Ophthalmic Hospital on having made so wise a choice for the post of matron.

### Notes and Queries.

To Correspondents.—1. Questions may be wri tten on post-cards. 2. Advertisements in disguise are inadmissible. 3. In answering a query please quote the number. 4. If a private answer is desired, a stamped addressed envelope must be forwarded.

(26) *Miss K.* will be very glad of any information regarding Nurses being sent to South Africa from an institution or through a lady.

(27) *Nursing the Insane.*—Coombe would be glad to know the names, prices, and publishers of books on the nursing of the insane.

(28) *Home for an Epileptic.*—A home wanted, either free or very small payment, for an incurable epileptic, aged 67. Midlands preferred,—*H. Austin.*

(29) *Books on Midwifery.*—Please tell me some good books to help me in the study of midwifery.—*Nurse T.*

(30) *A Home Wanted.*—A home of rest wanted for a poor over-worked servant girl with swollen legs. She has no means.—*M. P.*

(31) Would you kindly inform me where I can obtain a list of homes for cripples, or institutions which receive disabled persons? I am interested in the case of a little girl who has just lost both her legs, but who, properly trained, might make a living by her hands. Many friends have made a small subscription for her, and I am anxious to find her a home.—Arthur Fisher, Hon. Sec., Tiverton Infirmary.

### Answers.

(27) *Nursing the Insane.*—"The Nursing and Care of the Nervous and Insane," by Charles K. Mills, M.D., Edinburgh. Young, J., Pentland, 4s. 6d.

(29) *Books on Midwifery.*—"Antiseptic Midwifery," by Dr. Clement Godson, 15, Buckingham Street, Strand, 6d. "A Short Manual for Monthly Nurses," by Dr. Cullingworth Churchill, 1s. 6d.

(31) "Help in Sickness: Where to Go and What to Do," by Henry C. Burdett, published by Messrs. Kegan Paul and Trench, price 1s. 6d., will give all necessary information on the subject. The Home of Rest, Haggerston, mentioned on page 24, might take the case.

*Maternity Hospital.*—The Glasgow Maternity Hospital has 34 beds, nearly all constantly occupied.

*Nurse Finns.*—In reply to many correspondents we have written to inquire after Nurse Finns, but have not yet received an answer.

